# Comparative Analysis of Clothing Pressure Distribution in Obese and Normal-Weight Dogs Based on Material and Postural Variations Using CLO 3D Virtual Fitting

**DOI:** 10.3390/ani16071006

**Published:** 2026-03-25

**Authors:** Jisoo Kim, Youngjoo Chae

**Affiliations:** Department of Clothing & Textiles, Chungbuk National University, Cheongju 28644, Republic of Korea; jisu0122@naver.com

**Keywords:** obese dogs, clothing pressure distribution, 3D virtual fitting, material and postural variation, pet apparel

## Abstract

Clothing pressure influences the comfort, mobility, and welfare of dogs; however, quantitative evidence on how obesity affects localized garment pressure is limited. Using CLO 3D virtual fitting, we evaluated clothing pressure according to body condition (normal vs. obese), posture, and fabric type. We constructed normal and obese avatars for three breeds and simulated a short-sleeved T-shirt across six postures and three fabrics, yielding *n* = 108 simulation conditions (two body conditions × three breeds × six postures × three fabrics). Clothing pressure was quantified as ROI-averaged pressure (kPa) at four body regions (P1–P4). The overall mean pressure (averaged across P1–P4) increased from 16.69 ± 3.69 kPa (normal) to 19.56 ± 5.03 kPa (obese), with the highest pressures consistently observed at the chest (P2) and abdomen (P4). Region-specific ANOVA/GLM analyses (breed treated as a fixed factor) showed significant main effects of body condition, posture, fabric type, and breed on clothing pressure (all *p* < 0.001), while the three-way interaction (body condition × posture × fabric) was not significant (*p* > 0.05). These findings show that CLO 3D virtual fitting enables controlled, simulation-based comparisons of clothing pressure across body conditions; however, because no in vivo wear trials were conducted, the results should be interpreted as preliminary, and they require future experimental validation before practical application.

## Simple Summary

This study used a 3D virtual fitting system to examine how dog body condition, posture, and fabric type affect clothing pressure exerted by short-sleeved dog T-shirts. By comparing normal-weight and obese avatars of three dog breeds across multiple postures (held snapshots representing dynamic activities) and fabric conditions, the results showed that obese dogs generally experienced higher and more localized pressure, particularly on the chest and abdomen, and that pressure increased when stiffer fabrics were used. Overall, posture and fabric type had a stronger influence on pressure than body condition alone, suggesting the importance of apparel designs that account for posture-related fit, fabric properties, and body shape to enhance comfort, especially for heavier dogs.

## 1. Introduction

With growing interest in product design that considers the health and welfare of companion dogs, research aimed at quantitatively analyzing canine body characteristics and movement has expanded. Accordingly, body condition differences—particularly obesity-related changes that may influence garment fit, body–garment contact, and mechanical loading—are increasingly important to consider in the design and evaluation of functional products intended for everyday wear. Dog obesity is recognized as a major global health issue, involving not only weight gain but also physiological and biomechanical changes such as enlargement of chest and abdominal circumference, changes in body surface geometry that can affect garment contact, fit, and localized pressure concentration, and increased musculoskeletal and respiratory burden. According to the Association for Pet Obesity Prevention [1], approximately 59% of dogs in the United States were classified as overweight or obese as of 2023, and high obesity rates (40–55%) have also been reported in Europe and the United Kingdom. Obesity can negatively affect metabolic function, activity levels, and overall comfort in daily life [2].

Changes associated with obesity extend beyond appearance, altering how the body interacts with the external environment. Obese dogs tend to show reduced activity levels and greater physical strain during movement and postural transitions compared with dogs of normal body condition [3]. In addition, owner lifestyle and perceptions are closely linked to canine obesity [4]. Because feeding routines, exercise opportunities, and everyday handling practices are sustained over time, these lifestyle-related factors can contribute to persistent obesity prevalence, making obese dogs a realistic and recurring wearer group in home environments. Therefore, obesity should be considered a key body condition in functional product design rather than an atypical case.

In parallel, canine apparel has increased in popularity due to shifting cultural perceptions of companion animals, social media influence, and growing demand for products that combine functional and esthetic attributes. Beyond thermal insulation and protection, dog apparel must support comfort and freedom of movement during wear. Clothing pressure directly affects wear comfort and mobility and may relate to behavioral responses and welfare. While pressure garments have been reported to alleviate anxiety-related behaviors in some cases, excessive pressure can induce stress and physical discomfort [5,6,7]. Obese dogs may be more susceptible to localized pressure concentrations than normal-weight dogs when wearing identical clothing, yet systematic analyses addressing this issue remain limited.

To reduce ethical, temporal, and economic constraints associated with physical wear trials, 3D virtual fitting technology has been widely adopted in apparel research. Virtual fitting can simulate garment fit and deformation by implementing patterns and fabric properties in a digital environment based on wearer body data [8], enabling controlled manipulation of body shape and posture with improved reproducibility and comparability [9]. Among available tools, CLO 3D (version 2025.2.256, CLO Virtual Fashion Inc., Seoul, Republic of Korea) provides color-based pressure maps and numerical outputs, allowing simultaneous qualitative and quantitative analysis of garment fit and pressure distributions (pressure reported in kilopascals, kPa) [10,11]. CLO 3D has been used to examine pressure changes under different fabrics, body conditions, and postures in human apparel contexts [12,13]; however, quantitative clothing pressure analyses for companion-animal apparel, particularly under pronounced body condition differences such as obesity, remain scarce. Existing pet wear research has largely focused on design practices, wearing conditions, and consumer perceptions [14,15,16]. Studies examining wearable products have also shown that items such as harnesses can affect canine gait and joint motion during walking [17]. In addition, animal behavioral research has reported that everyday dog postures and movements (e.g., walking, sitting, lying down, and body shaking) are associated with physiological and behavioral responses [18,19,20,21,22]. However, what remains missing is a region-specific, quantitative characterization of clothing pressure distributions that jointly considers body condition (normal vs. obese), posture, and fabric properties—i.e., how pressure at key body regions changes across these combined factors under realistic movement/posture scenarios. Addressing this gap is important for evaluating potential localized pressure concentration in obese dogs and for establishing an evidence base for simulation-informed design and assessment.

Accordingly, this study aimed to comparatively analyze clothing pressure distributions in dogs of normal and obese body conditions using CLO 3D virtual fitting. We constructed normal and obese avatars for three representative breeds (Bulldog, Labrador retriever, and German shepherd) and applied a short-sleeved T-shirt-type garment to evaluate pressure at four body regions (neck, chest, back, and abdomen) across combinations of body condition, fabric type, and posture. By systematically comparing these conditions in a controlled simulation environment, this study provides foundational data for functional pet wear design that accounts for diverse body conditions, including obesity, while reducing the practical constraints of physical wear trials and expanding virtual fitting approaches in pet wear research.

## 2. Materials and Methods

### 2.1. Three-Dimensional Virtual Try-On Setup

#### 2.1.1. Dog Avatars and Body Shape Conditions

Three representative dog breeds with distinctly different body shapes were selected to comparatively analyze clothing pressure distributions according to dogs’ body shape conditions. The selected breeds were the Bulldog, Labrador retriever, and German shepherd, which are commonly kept in Europe and the United States [23,24]. Importantly, the primary rationale for breed selection in this study was to represent distinct thoracic/body shape types relevant to garment fit and pressure concentration rather than to treat breeds as “replicates” or to broadly represent all dog sizes. Specifically, the Bulldog represents a barrel-chested, brachycephalic low-stature morphology with a broad thorax and comparatively short trunk; the Labrador retriever represents a round-/broad-chested, balanced morphology with relatively uniform girth distribution across the trunk; and the German shepherd represents a deep-chested, athletic morphology with a pronounced thoracic depth and a more tapered waist. This morphology-based selection was intended to capture body shape diversity that can influence pattern fit and localized clothing pressure distribution in short-sleeved T-shirt-type dog apparel.

Normal and obese conditions were established for each breed to construct six dog avatars. Normal avatars were implemented in CLO 3D based on standard breed proportions. Because CLO 3D offers limited control over localized body volume changes, obese avatars were generated in Blender (version 5.0; Blender Foundation, Amsterdam, the Netherlands) by editing the surface mesh of the corresponding normal avatars while preserving back length and the original rigging. Obesity was parameterized by matching key circumferential landmarks (neck, chest, and abdomen) to the obese condition measurements in Table 1. To support biological plausibility, volume increases were applied primarily to the thoracoabdominal trunk rather than via uniform global scaling, while maintaining skeletal landmarks and joint articulation. The modified avatars were exported as FBX and re-imported into CLO 3D. For reproducibility, before/after landmark measurements and percent changes are provided in Supplementary Table S4.

The obese body condition established in this study was based on a clinically common moderate obesity level (body condition score (BCS) of approximately 7 on the 1–9 point scale), which is defined as in the range between overweight and moderate obesity [2,25]. The obese avatars were created by enlarging the body volume primarily in the chest, abdomen, hips, and upper shoulders, which are areas where body volume increments are typically observed in obese dogs, while maintaining similar skeletal structures, body length proportions, and rigging as those in the normal body condition. Moreover, while maintaining the distinct body characteristics of each breed, the degree of body fat increment relative to the normal condition was controlled at similar levels across all breeds to analyze the effect of obesity on changes in clothing pressure. The created obese avatars were converted to the FBX format and imported into the CLO 3D environment, where they were used under similar simulation conditions as those of the normal avatars. The key body measurements for each avatar are listed in Table 1.

#### 2.1.2. Garment Design and Material Conditions

**Figure 1:**
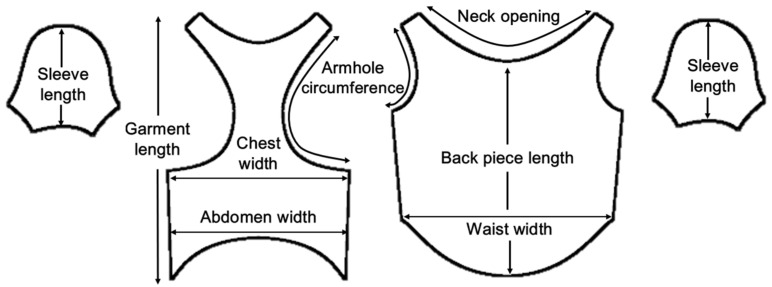
T-shirt-type garment pattern for dog apparel.

The garment applied in this study was a short-sleeved T-shirt-type design that is frequently sold in the dog apparel market. Short-sleeve T-shirts are widely used as a basic form for both daily and functional garments, regardless of season [14,15], and were considered to be suitable for comparatively analyzing the differences in clothing pressure distributions according to body condition and postural variations in dogs. A basic T-shirt pattern without decorative elements was applied, and similar pattern structures and seam configurations were maintained under all experimental conditions. The pattern dimensions for each body condition were proportionally adjusted to match body measurements of each avatar. The pattern structure and key dimensional items are presented in Figure 1 and Table 2.

Three fabric types were selected: polyester–spandex, cotton–spandex, and polar fleece, to reflect the common blends used in the dog apparel market [14,15]. The cotton–spandex fabric was a 95% cotton/5% spandex blend to represent everyday T-shirt-type pet wear, whereas the polyester–spandex fabric was a 90% polyester/10% spandex blend to reflect the characteristics of functional pet wear for walking and active use. The polar fleece fabric was 100% polyester fleece, which is widely used in thermal pet wear, representing fabric characteristics with relatively high thickness and stiffness with limited stretch.

Fabric property inputs (areal density, thickness, stretch parameters, and friction coefficient) were obtained from the built-in CLO 3D fabric library presets and used as simulation inputs without additional calibration (Table 3). These values were not measured from physical fabric specimens in this study; therefore, the parameters in Table 3 should be interpreted as preset-based inputs intended for controlled comparative analysis rather than as product-specific, experimentally validated material measurements. Accordingly, absolute clothing pressure values should be understood within the simulation framework, and measurement-based calibration/validation remains an important direction for future work.

#### 2.1.3. Posture Conditions and Simulation Settings

Virtual fitting simulations were conducted for six representative postural conditions that are frequently observed in the daily activities of dogs. The selected postures included walking, sitting, lying down, running, forelimb stretching, and unilateral hind limb lifting, representing both static poses and dynamic movements. Table 4 shows representative examples to explain the different posture conditions; similar posture definitions and simulation procedures were applied across all three breeds. These postures represent the daily activities of dogs and have been reported to produce relatively pronounced changes in shape and garment deformation of the trunk, chest, abdomen, and limbs [18,26].

All posture conditions were implemented as static poses (snapshots) in the CLO 3D environment to enable controlled comparisons across body condition, fabric type, and breed. Postures described as “dynamic” activities (e.g., running and unilateral hind limb lifting) therefore represent held poses at representative points in the movement cycle rather than time-resolved motion sequences; accordingly, the effects of movement velocity and trajectory are not modeled in the present simulations.

All simulations were conducted in CLO 3D (version 2025.2.256). Scene-level simulation properties and solver/physics settings were maintained consistently across all conditions. Unless otherwise stated, CLO default global settings were used. To ensure sufficient mesh resolution for pressure map inspection and ROI-based metrics, the particle distance was set to 20 mm during garment setup and then reduced to 5 mm (hi-res garment; complete quality) before the final simulation and pressure extraction. Collision handling parameters were also fixed across conditions: the garment simulation thickness (collision thickness) was set to 1.5 mm on each side (total 3 mm), and the avatar skin offset was set to 3 mm to prevent interpenetration during simulation. No formal sensitivity analysis of CLO 3D simulation parameters was conducted; however, all conditions were simulated under identical settings to ensure internally consistent comparisons. To ensure comparability of ROI-averaged pressure values across breeds and postures, mesh resolution was kept consistent under the same CLO 3D simulation settings (particle distance fixed at 5 mm for final simulations), and ROI-averaged pressure was computed using an identical ROI definition protocol across all conditions.

Each simulation was analyzed only after a stable configuration was reached. The stability threshold was selected based on pilot simulations, in which pressure time-series fluctuations became negligible beyond this point; quantitative outputs were exported only after meeting the stability criterion to minimize transient effects. Stability (convergence) was defined operationally as: (i) no visible oscillation of the garment on the avatar and (ii) negligible residual motion maintained for at least 3 s, during which (iii) ROI-averaged clothing pressure values changed minimally (≤1%) across consecutive checks. Clothing pressure was extracted from the final stabilized state for subsequent qualitative (pressure map) and quantitative (ROI-based) analyses. Quantitative kPa outputs served as the primary analytical basis, while color maps were provided for visualization only.

CLO 3D has been developed and validated primarily for human garments; therefore, canine-specific validation of absolute clothing pressure outputs remains limited. Because this study did not include calibration or validation against pressure measurements on live dogs, the absolute kPa values should be interpreted cautiously and used primarily as relative indicators for controlled comparisons across body condition, posture, and fabric type.

### 2.2. Clothing Pressure Measurement

The clothing pressure data were extracted using the pressure map function provided by the CLO 3D system. CLO 3D represents the pressure generated by the interactions between the garment and body surface in color-based pressure maps and numerical data, enabling a simultaneous analysis of the spatial distribution and magnitude of clothing pressure.

**Figure 2:**
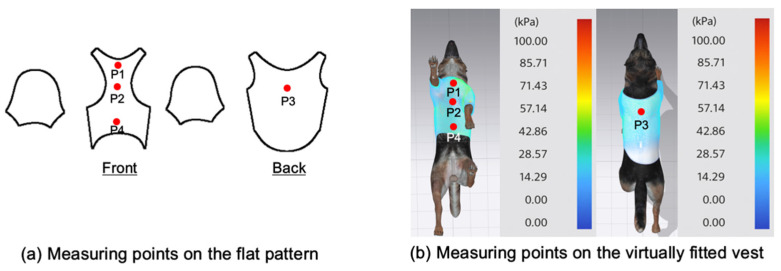
Pressure measurement regions (ROIs) used for quantitative extraction: P1 (neck), P2 (chest), P3 (back), and P4 (abdomen/ventral side).

This study selected four body regions (i.e., the neck, chest, back, and abdomen), where pressure concentration was expected during dog apparel wear, as measurement regions (regions of interest; ROIs) (Figure 2). In the flat-pattern and fitted views, P1–P2 are indicated on the front view, P3 on the back view, and P4 on the ventral (abdominal) region, which may not be fully visible in a purely frontal projection. Each measurement location was defined as an ROI based on consistent anatomical landmarks, and the reported value represents the mean pressure within the ROI (kPa) obtained from CLO 3D’s pressure output rather than a single mesh vertex. Specifically, P1 (neck) was defined around the base of the neck, near the neckline/collar region; P2 (chest) was defined at the ventral thoracic region around the level of maximum chest girth (sternum level); P3 (back) was defined along the dorsal midline on the trunk (mid-back region); and P4 (abdomen) was defined at the ventral abdominal region around the level of maximum abdominal girth (cranial to the pelvis). ROIs were positioned using the same landmark-based criteria across all breeds and posture conditions to ensure consistent placement.

In the pressure map, blue indicates low pressure, green and yellow indicate medium pressure, and red indicates high pressure. The qualitative analysis was conducted by visually comparing the color distribution of the pressure maps, whereas the quantitative analysis involved extracting ROI-averaged clothing pressure values for P1–P4. All pressure values were standardized in kilopascals.

This study did not involve physical wear trials on live dogs; rather, it aimed to explore the characteristics of pressure distributions according to body and postural conditions using clothing pressure analysis based on 3D virtual fitting. This research design considered potential stress and ethical issues that may arise in animal experiments or trials and can be applied as foundational data for future validation studies involving actual wear trials.

### 2.3. Data Analysis

**Figure 3:**
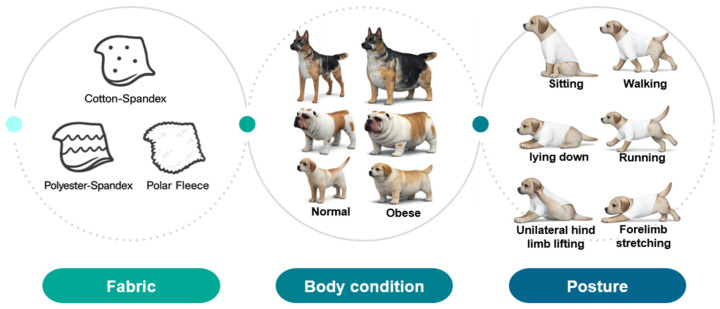
Experimental design and analysis conditions for clothing pressure evaluation.

In this study, CLO 3D virtual fitting was conducted under 108 simulation conditions comprising combinations of body conditions (normal and obese), breeds (three types), postures (six types), and fabrics (three types) (Figure 3). The qualitative and quantitative analyses of the clothing pressure on the neck (P1), chest (P2), back (P3), and abdomen (P4) under each condition were conducted. For the qualitative analysis, the CLO 3D pressure map was used to visually compare and interpret the patterns of pressure distribution and localized pressure concentration areas according to body condition, posture, and fabric type. For the quantitative analysis, clothing pressure values extracted from each body part (P1–P4) were compared under different conditions.

The statistical analyses were performed using the SPSS software (version 28.0; IBM Corp., Armonk, NY, USA). Breed was treated as a fixed factor to avoid pseudo-replication, and inferential analyses were conducted separately for each body region (P1–P4). For each region, a factorial ANOVA/GLM (univariate GLM in SPSS) was fitted including the main effects of body condition (two levels), posture (six levels), fabric (three levels), and breed (three levels), as well as interaction terms (body condition × posture, body condition × fabric, posture × fabric, and body condition × posture × fabric). The significance level was set at α = 0.05, and Tukey’s HSD was used for post hoc pairwise comparisons where appropriate. Model assumptions were assessed using residual diagnostics (Q-Q plots/Shapiro-Wilk test for normality; Levene’s test for homogeneity of variances).

## 3. Results and Discussion

### 3.1. Qualitative Assessment of Clothing Pressure Distribution

This study qualitatively analyzed the characteristics of clothing pressure distributions of short-sleeved T-shirt-type dog apparel according to the body condition, posture, and fabric type, using the pressure map function of the CLO 3D virtual fitting system. The qualitative pressure map patterns were broadly consistent across breeds; therefore, a representative breed is shown in the main text (Table 5), and the corresponding tables for the remaining breeds are provided in the Supplementary Materials (Tables S5 and S6). Table 5 presents the clothing pressure distribution across six postures for each fabric under normal and obese body conditions, shown from the front, top, and bottom views.

Overall, the clothing pressure distribution patterns tended to differ according to body condition in all three breeds, and changes associated with body condition (normal versus obese) were more pronounced than differences between breeds under the conditions set in this study. In dogs with a normal body condition, low-pressure areas in the blue–green range were relatively widely distributed across the back and sides for different postures and fabric types. Particularly, the garments did not cling excessively to the body during static postures (sitting and lying down), resulting in low- or medium-pressure distributions. In contrast, for obese dogs, areas in green–yellow or red colors were extensively observed in the trunk region (chest and abdomen), even when the patterns and fabric types were identical, and high-pressure areas in the yellow–red range were more clearly observed in certain conditions. This can be interpreted as a result of increased body volume in the trunk because of weight gain, causing the garment to fit more tightly to the body surface, thereby resulting in concentrated local tension.

When comparing postures, both normal and obese body conditions tended to show color distributions consistent with relatively higher pressures in dynamic postures, such as running and unilateral hind limb lifting. Particularly, for obese body conditions, these postures often exhibited more extensive medium- or high-pressure areas (e.g., yellow or orange–red) in the trunk region or more clearly defined high-pressure areas (orange–red), implying differences in pressure sensitivity according to the body condition. Conversely, during static postures (sitting and lying down), both body conditions showed relatively mild distributions; however, the medium-pressure areas were more extensive in obese dogs than in normal dogs.

When examining clothing pressure distributions according to fabric type, the color distribution indicating the highest pressure range (orange–red) was predominantly observed in polar fleece, with a gradual reduction in pressure levels for polyester–spandex and cotton–spandex. Particularly, the polar fleece T-shirt frequently exhibited repeated high-pressure areas in the trunk region of obese dogs, suggesting that clothing pressure can increase under the combined effects of body condition and fabric characteristics. Conversely, spandex-blend fabrics with high stretchability showed relatively moderate distributions in normal dogs, whereas in obese dogs, the increased body volume caused the garment to fit more tightly, resulting in medium or high pressure persisting in certain body parts.

In summary, the qualitative analysis using the CLO 3D pressure map visually demonstrated that pressure concentration can occur in the trunk region of obese dogs, even when similar garments are used. However, although the pressure map is useful as a color-based visualization tool for identifying relative trends in pressure distributions, similar pressure values may be perceived differently depending on the body condition, body surface curvature, or viewing angle. Thus, a qualitative analysis is essential for identifying the distribution trends, and the results must be integrated with those of the quantitative analysis presented in the next section (Table 6) for a more rigorous comparison and interpretation.

### 3.2. Quantitative Comparison of Clothing Pressure Levels

#### 3.2.1. Overview of Quantitative Analysis and Variables

The clothing pressure distributions of short-sleeved T-shirt-type garments on normal and obese dogs were first examined using CLO 3D color pressure maps. While this qualitative visualization is useful for identifying relative distribution trends, it is limited for precise comparisons of pressure magnitude across body conditions and for objectively quantifying the effects of posture and fabric type.

Therefore, we quantitatively analyzed CLO 3D-derived pressure values to evaluate the effects of body condition (normal vs. obese), posture (walking, sitting, lying down, running, forelimb stretching, and unilateral hind limb lifting), and fabric type (cotton–spandex, polyester–spandex, and polar fleece). ROI-based pressures were assessed with particular attention to the chest (P2) and abdomen (P4), which are closely related to wear comfort. Table 6 summarizes the clothing pressure values obtained from the virtual fitting simulations.

#### 3.2.2. Comparison of Clothing Pressure Levels Based on Body Condition and ROIs

**Figure 4:**
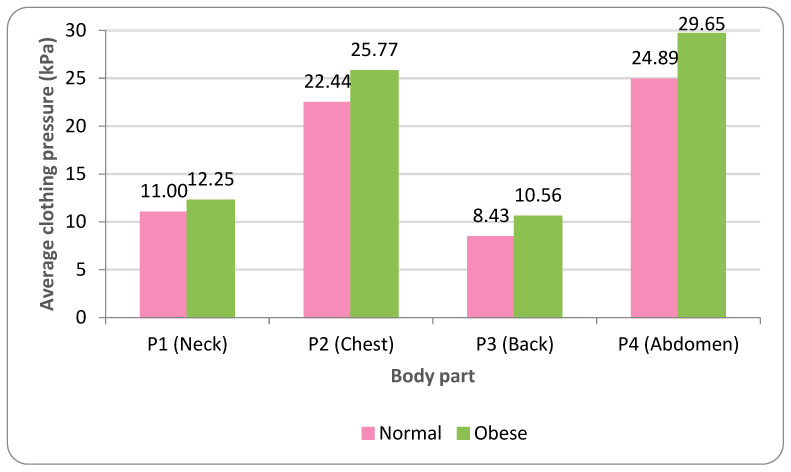
Average clothing pressure by ROI (P1–P4) under normal and obese body conditions (unit: kPa). Note: values are mean ROI-averaged pressures for each region, averaged across three breeds, six postures, and three fabric types (see Table 6).

Based on the overall mean clothing pressure (averaged across P1–P4), normal and obese dogs exhibited 16.69 ± 3.69 kPa and 19.56 ± 5.03 kPa, respectively, corresponding to an average increase of 2.87 kPa under obesity (Table 6; Figure 4). Across ROIs, the abdomen (P4) and chest (P2) consistently showed the highest pressures, whereas the neck (P1) and back (P3) were lower under both body conditions. In normal dogs, mean pressures were 24.89 kPa (P4) and 22.44 kPa (P2), compared with 11.00 kPa (P1) and 8.43 kPa (P3); the same rank order was observed in obese dogs (P4: 29.65 kPa; P2: 25.77 kPa; P1: 12.25 kPa; P3: 10.56 kPa) (Table 6; Figure 4). Obesity-related increases were the greatest at the thoracoabdominal ROIs, with P4 and P2 increasing by 4.76 kPa (24.89 → 29.65 kPa) and 3.33 kPa (22.44 → 25.77 kPa), respectively, whereas smaller increases were observed at P1 and P3 (1.25 and 2.13 kPa). This pattern may reflect obesity-related enlargement and surface geometry changes at the chest and abdomen, which could increase garment contact and localized tension in these regions.

By contrast, the neck (P1) and back (P3) exhibited lower pressures, consistent with their comparatively gentler surface geometry and greater allowance for load distribution during wear. These findings align with prior reports that obesity increases mechanical loading associated with trunk enlargement [2] and that the same wearable device can exert higher pressure and restrict movement on specific body parts as body size increases [17]. Together, our simulation-based results provide quantitative support for these observations under controlled virtual fitting conditions.

#### 3.2.3. Clothing Pressure Characteristics Based on Posture and ROIs

Comparisons by posture showed that obese dogs consistently experienced medium or higher clothing pressure than normal dogs, even in static postures (sitting and lying down). This difference was further amplified in held snapshot postures of dynamic activities (e.g., running and unilateral hind limb lifting). The mean difference (Δ) between obese and normal dogs was 1.36 kPa in static postures, whereas it increased to 4.27 kPa in snapshot postures (Table 6). While color pressure maps support qualitative visualization, map appearance can vary with curvature and viewing angle; therefore, the interpretations were supported primarily by quantitative ROI-based kPa comparisons.

Among all postures, running and unilateral hind limb lifting yielded the highest overall pressures under both body conditions. As shown in Table 6 (mean across P1–P4), running and unilateral hind limb lifting produced 19.52/19.05 kPa in normal dogs and 23.62/23.48 kPa in obese dogs, respectively. Pressure increases were concentrated at the thoracoabdominal ROIs. In obese dogs, the abdomen (P4) showed high pressures during unilateral hind limb lifting and running (36.26 and 35.72 kPa), and the chest (P2) was also elevated (31.04 and 30.58 kPa). These patterns may be associated with increased thoracoabdominal deformation in these postures, potentially contributing to localized garment tension at the chest and abdomen.

In the context of canine welfare, external compression from worn products has been discussed most often in relation to pressure garments/wraps used for situational anxiety. In some studies, moderate torso compression has been examined with measurable physiological or behavioral changes (e.g., reduced heart rate increase under a fitted pressure wrap), although reported effects can be variable and context-dependent [6,7]. Overall, these findings highlight the importance of considering not only target ROIs but also posture conditions encountered during wear.

#### 3.2.4. Differences in Clothing Pressure by Fabric Type and Interpretation of CLO 3D Simulations

**Figure 5:**
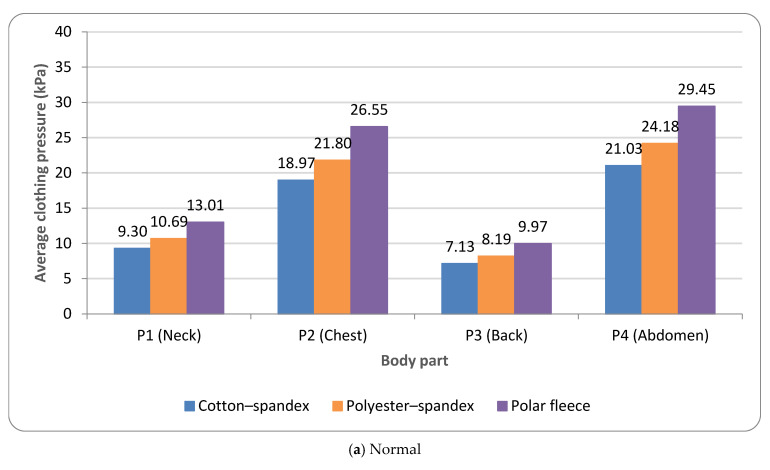

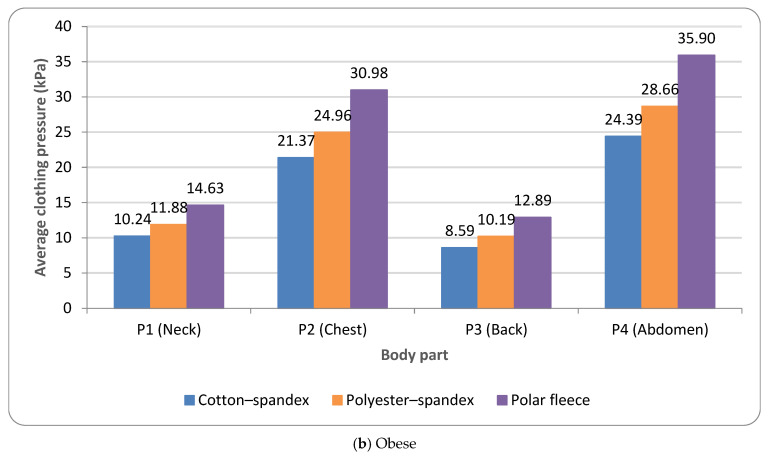
Average clothing pressure by fabric type under normal and obese body conditions (unit: kPa). Note: values are ROI-averaged pressures for each fabric type, averaged across three breeds, six postures, and four ROIs (P1–P4) (see Table 6).

A comparison by fabric type showed that polar fleece produced the highest clothing pressure, followed by polyester–spandex and cotton–spandex (Table 6; Figure 5). Based on the overall mean pressure (averaged across ROIs, breeds, and postures), cotton–spandex, polyester–spandex, and polar fleece yielded 14.11, 16.22, and 19.75 kPa in normal dogs, respectively; the same order was observed in obese dogs (16.15, 18.92, and 23.60 kPa). Notably, the pressure gap among fabrics was larger under obesity (polar fleece vs. cotton–spandex: 5.64 kPa in normal dogs vs. 7.45 kPa in obese dogs), suggesting that increased body volume may amplify fabric-dependent differences in garment–body contact.

Polar fleece, which is relatively thicker and stiffer, generated particularly high pressures at the thoracoabdominal ROIs in obese dogs. Under polar fleece, obese dogs showed markedly elevated pressures at the abdomen (P4: 35.90 kPa) and chest (P2: 30.98 kPa) compared with cotton–spandex (P4: 24.39 kPa; P2: 21.37 kPa) and polyester–spandex (P4: 28.66 kPa; P2: 24.96 kPa). By contrast, spandex-blended fabrics maintained lower overall pressures, although the buffering effect under obesity appeared reduced, contributing to an expanded pressure gap between fabric types.

Posture and fabric type also interacted to increase pressure in specific conditions. For example, in the running + polar fleece condition, mean pressures across breeds were 23.09 kPa (normal) and 28.51 kPa (obese), indicating a larger increase under obesity. Together, these results suggest that obese dogs may experience greater pressure variability across wearing conditions (posture × fabric), even when garment design is held constant.

Finally, quantitative outputs provided a more nuanced view than pressure maps alone. While pressure maps visually accentuated localized high-pressure areas, numerical outputs showed that pressure levels varied continuously across posture and fabric conditions. Thus, pressure maps are useful for visualizing relative distribution patterns, but robust interpretation requires quantitative ROI-based comparisons (e.g., Table 6), particularly when comparing body conditions and fabrics.

#### 3.2.5. Effects of Body Condition, Posture, Fabric, and Breed on Clothing Pressure (Region-Specific Analysis)

Based on the clothing pressure data extracted from the CLO 3D simulations, we conducted ROI-specific inferential analyses to evaluate the effects of body condition (normal vs. obese), posture, fabric type, and breed on clothing pressure. To avoid pseudo-replication, breed was treated as a fixed factor, and analyses were performed separately for each ROI (P1–P4) rather than using a single averaged outcome. The results for chest pressure (P2) are summarized in Table 7, and corresponding outputs for P1 (neck), P3 (back), and P4 (abdomen) are provided in Supplementary Tables S1–S3.

Across ROIs, fabric type, posture, body condition, and breed showed statistically significant main effects on clothing pressure (all *p* < 0.001; Table 7 and Tables S1–S3). In addition, two-way interactions (body condition × fabric, fabric × posture, and body condition × posture) were statistically significant across ROIs (all *p* < 0.001), indicating that the influence of material properties and postural deformation on pressure depends, in part, on body condition and vice versa. By contrast, the three-way interaction (body condition × fabric × posture) was not statistically significant across ROIs (*p* = 0.661 for P2; *p* = 0.366 for P1; *p* = 0.640 for P3; *p* = 0.730 for P4), suggesting that higher-order combined effects were limited within the scope of the present simulation design.

Overall, these findings support the quantitative comparisons reported above and indicate that clothing pressure patterns in the CLO 3D-based virtual fitting environment are jointly influenced by body condition, posture, fabric properties, and breed-related morphology, with interaction effects present primarily at the two-way level. For clarity, an earlier version of the manuscript reported a three-way ANOVA based on a single aggregated outcome (mean pressure averaged across P1–P4). In the revised manuscript, inferential results are reported using ROI-specific models (P1–P4 analyzed separately), with breed treated as a fixed factor (Table 7 and Tables S1–S3); therefore, SS/F/df/*p*-values are not directly comparable to those from the aggregated analysis. Thus, the ROI-specific models (P1–P4) provide the primary inferential basis for interpreting body condition effects in this study.

These ROI-specific effects may have implications for comfort and mobility during wear, within the scope of the simulation-based findings. Beyond anxiety-focused garments, studies on canine wearable equipment show that harness design can alter locomotion and restrict joint range of motion (e.g., reduced shoulder extension and changes in stride-related kinematics), highlighting that fit and contact mechanics of worn products can have functional implications for movement and comfort [27,28,29].

### 3.3. Future Directions and Recommendations for Software Development (Conceptual; Not Implemented in This Study)

CLO 3D (version 2025.2.256) has been developed primarily for human wearers; thus, limitations remain when applying it to canine bodies with greater morphological diversity and body condition-related shape changes. Based on the region-specific pressure patterns observed in this study, we outline the following conceptual recommendations for future software development. These features are not implemented or empirically tested in the present work and would require development and validation.

**Figure 6:**
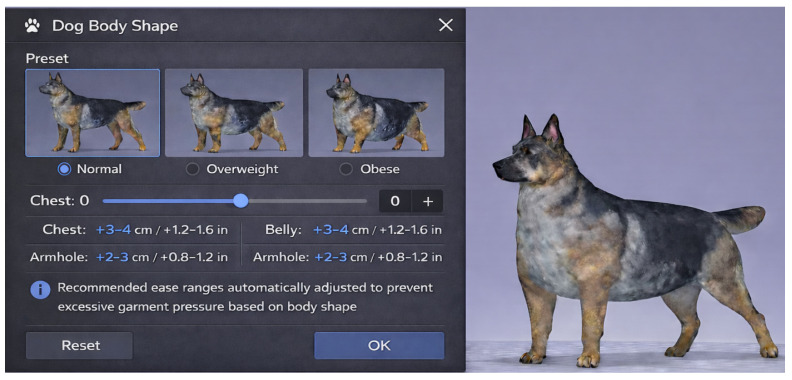
Proposed option 1: adjustable body size control for virtual dog avatars in CLO 3D.

**Figure 7:**
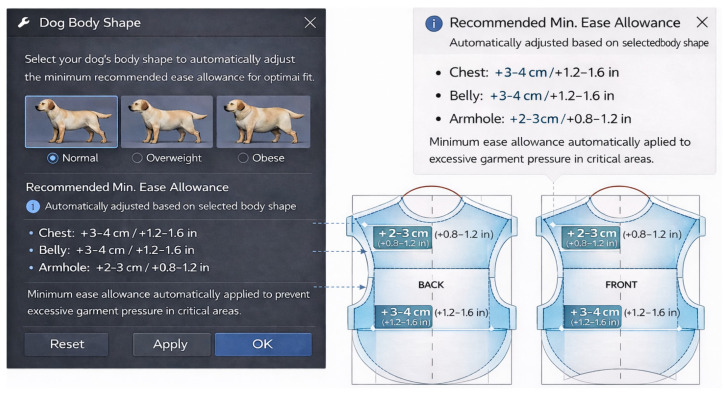
Proposed option 2: automated ease allowance guidance according to canine body size.

**Figure 8:**
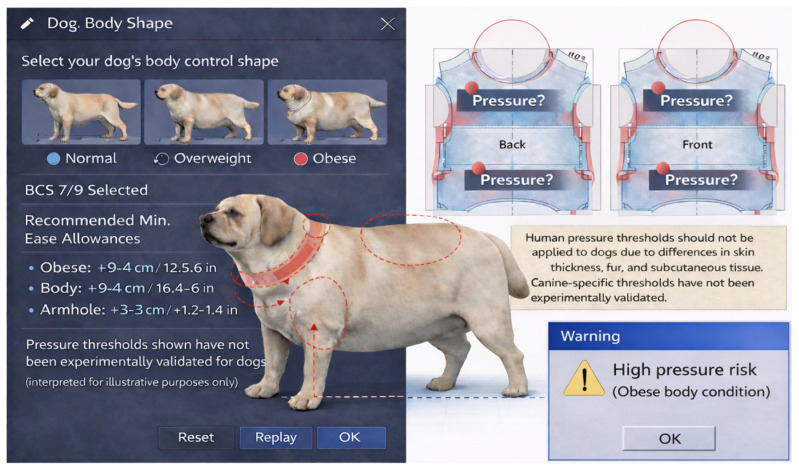
Proposed option 3: conceptual interface for highlighting comparatively high-pressure zones (illustrative; thresholds require validation).

**Figure 9:**
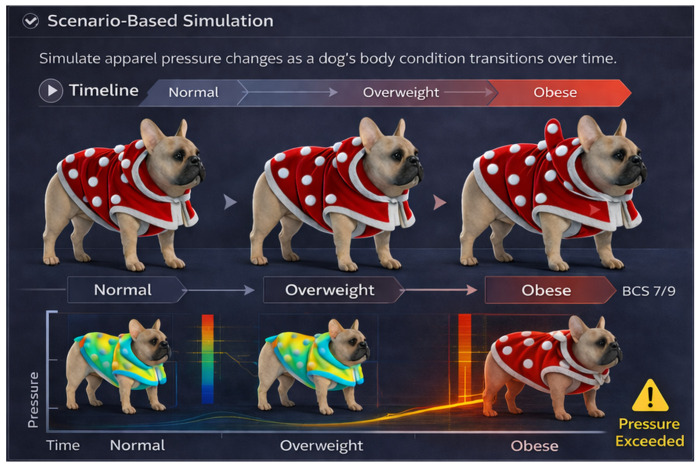
Proposed option 4: scenario-based simulation of progressive body condition changes.

(1) BCS-informed canine avatar parameterization. CLO 3D does not provide dog avatars or direct body condition controls; therefore, obese avatars in this study required external modeling (Blender), which limits efficiency and repeatability. Future platforms could provide dog-specific avatars with selectable body condition categories (e.g., normal/overweight/obese) and slider-based control of key regions (e.g., chest and abdomen) to support reproducible comparisons (Figure 6).

(2) Region-specific ease-allocation guidance. Because pressure increases concentrated in trunk regions (chest/abdomen/armhole) even under identical pattern structures, tools that suggest minimum ease ranges by body condition and region could reduce trial-and-error during pattern development (Figure 7).

(3) Interface for highlighting comparatively high-pressure zones (illustrative). Pressure maps are visually informative, but canine-specific thresholds for discomfort/welfare risk are not established. Rather than a fixed “risk” warning, an interface could flag comparatively high-pressure zones using a user-defined (provisional) criterion, contingent on future validation (Figure 8).

(4) Scenario-based simulation across progressive body condition change. CLO 3D currently supports static evaluation at a single body condition. Scenario-based simulations that track pressure while gradually changing body condition (normal → overweight → obese) could help explore how pressure patterns evolve under consistent garment conditions (Figure 9).

Overall, these items are presented as future directions motivated by the current findings and should be interpreted as recommendations requiring implementation and measurement-based validation before practical deployment.

## 4. Conclusions

This study analyzed clothing pressure distribution in short-sleeved T-shirt-type dog apparel across body condition (normal vs. obese), posture, and fabric type using CLO 3D virtual fitting. Six dog avatars (three breeds; normal/obese) were evaluated under six postures and three fabrics (108 conditions), and pressure was quantified at the neck (P1), chest (P2), back (P3), and abdomen (P4).

1. Pressure maps and ROI-based metrics indicated broader medium-to-high pressure regions in the obese condition, particularly at the chest (P2) and abdomen (P4). These patterns were more pronounced in held snapshot postures representing dynamic activities (e.g., running and unilateral hind limb lifting).
2. Mean pressure averaged across P1–P4 increased from 16.69 kPa (normal) to 19.56 kPa (obese). The largest increases were observed at the thoracoabdominal ROIs: abdomen (P4) increased from 24.89 to 29.65 kPa, and chest (P2) increased from 22.44 to 25.77 kPa.
3. Across postures, the abdomen (P4) and chest (P2) consistently showed the highest pressures, whereas the neck (P1) and back (P3) were lower (normal: P4 24.89 kPa, P2 22.44 kPa vs. P1 11.00 kPa, P3 8.43 kPa; obese: P4 29.65 kPa, P2 25.77 kPa vs. P1 12.25 kPa, P3 10.56 kPa). Running and unilateral hind limb lifting yielded the highest average pressures (normal: 19.52/19.05 kPa; obese: 23.62/23.48 kPa), with peak concentrations observed at the abdomen (e.g., 36.26 kPa at P4).
4. Fabric effects followed polar fleece > polyester–spandex > cotton–spandex. The average pressures were 14.11/16.22/19.75 kPa (normal) and 16.15/18.92/23.60 kPa (obese) for cotton–spandex/polyester–spandex/polar fleece, respectively. The differences among fabrics were larger under obesity, and polar fleece produced the highest thoracoabdominal pressures in obese dogs (P4 35.90 kPa; P2 30.98 kPa).
5. ROI-specific ANOVA/GLM analyses treating breed as a fixed factor showed significant main effects of body condition, posture, fabric type, and breed across ROIs (Table 7 and Tables S1–S3). Significant two-way interactions were observed, whereas three-way interactions (body condition × fabric × posture) were not significant, indicating that interaction effects were primarily expressed at the two-way level in the present design.

CLO 3D enabled controlled, repeatable comparisons without live wear trials; however, the simulations do not capture biological factors such as fur-related interface effects (e.g., coat length/density), soft-tissue compliance, or physiological/behavioral responses that may influence contact pressure. In addition, the present analysis focused on three medium-to-large breeds selected to cover distinct trunk morphologies and did not include toy breeds; because markedly different body proportions in toy breeds may require size-specific grading and rigging, the generalizability of the current findings to toy breeds is limited. Accordingly, absolute kPa values should be interpreted as comparative outputs within the simulation framework rather than validated clinical or physiological thresholds. Future work should include measurement-based validation under ethically appropriate designs and broaden coverage to additional breed/size groups (including toy breeds, e.g., Chihuahua and Pomeranian) and finer body condition stages beyond a binary normal/obese classification.

## Figures and Tables

**Table 1:** The sizes of avatars used in this study (unit: cm).

Dog Breed	Body Condition	Body Size			
		Neck Circumference	Chest Circumference	Abdomen Circumference	Back Length
Bulldog	Normal	42.0	68.0	65.0	38.0
	Obese	50.0	85.0	90.0	38.0
Labrador retriever	Normal	55.0	78.0	75.0	55.0
	Obese	65.0	100.0	105.0	55.0
German shepherd	Normal	52.0	80.0	76.0	58.0
	Obese	62.0	102.0	108.0	58.0

**Table 2:** Garment pattern dimensions according to dog breed and body condition (unit: cm).

Dog Breed	Body Condition	Garment Measurements					
		Garment Length	Back Piece Length	Chest Width	Abdomen Width	Neck Opening	Armhole Circumference
Bulldog	Normal	34.0	36.5	43.6	37.3	32.1	59.3
	Obese	34.0	36.5	47.6	42.9	36.9	66.3
Labrador retriever	Normal	51.0	53.5	50.0	43.0	42.0	68.0
	Obese	51.0	53.5	56.0	50.0	48.0	78.0
German shepherd	Normal	54.0	56.5	46.0	40.0	46.0	66.0
	Obese	54.0	56.5	54.0	52.0	52.0	80.0

**Table 3:** Physical properties of fabrics used in canine T-shirt simulation (unit: as indicated).

Fabric Type	Density (g/m^2^)	Thickness (mm)	Stretch (%)	Friction Coefficient
Cotton–spandex	180	0.60	10	0.30
Polyester–spandex	100	0.35	30	0.25
Polar fleece	350	1.50	5	0.40

**Table 4:** Posture conditions of virtual dog avatars under normal and obese body conditions.

Posture	Normal			Obese		
	Front	Side	Back	Front	Side	Back
Sitting	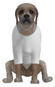	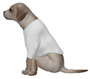	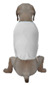	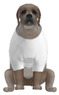	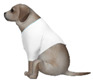	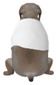
Walking	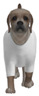	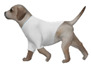	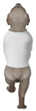	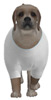	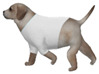	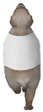
Lying down	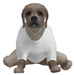	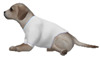	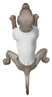	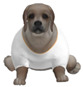	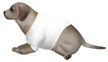	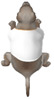
Running	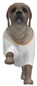	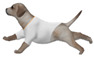	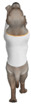	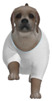	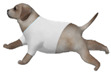	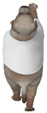
Forelimb stretching	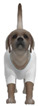	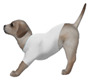	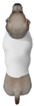	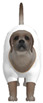	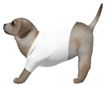	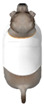
Unilateral hind limb lifting	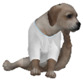	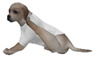	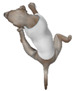	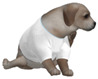	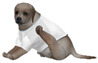	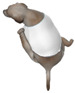

**Table 5:** Qualitative clothing pressure maps for the Labrador retriever across body condition (normal vs. obese), posture, and fabric type (front, top, and bottom views).

Fabric	Posture	Normal			Obese		
		Front	Top	Bottom	Front	Top	Bottom
Cotton–spandex	Sitting	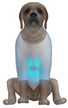	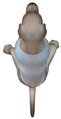	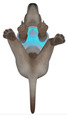	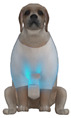	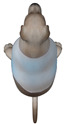	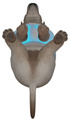
	Walking	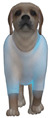	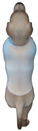	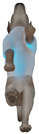	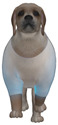	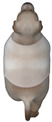	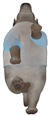
	Lying down	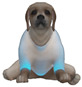	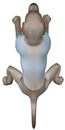	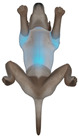	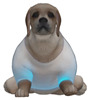	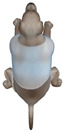	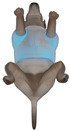
	Running	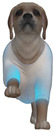	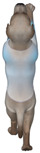	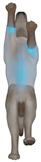	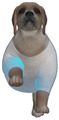	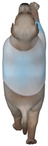	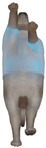
	Forelimb stretching	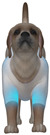	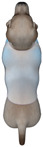	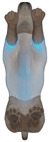	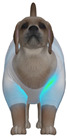	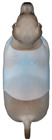	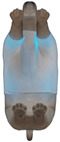
	Unilateral hind limb lifting	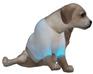	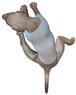	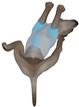	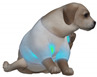	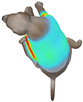	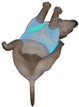
Polyester–spandex	Sitting	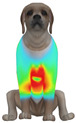	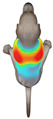	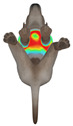	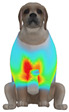	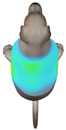	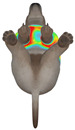
	Walking	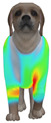	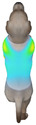	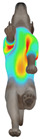	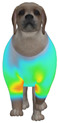	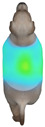	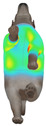
	Lying down	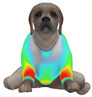	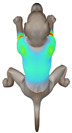	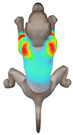	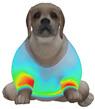	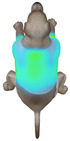	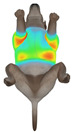
	Running	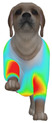	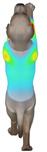	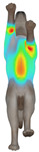	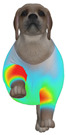	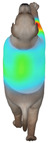	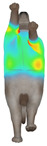
	Forelimb stretching	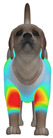	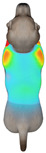	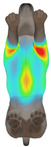	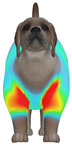	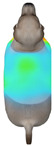	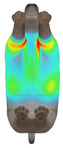
	Unilateral hind limb lifting	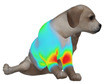	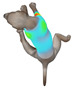	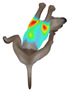	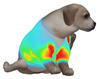	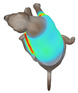	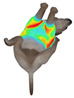
Polar fleece	Sitting	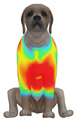	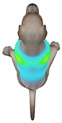	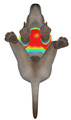	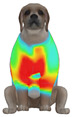	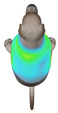	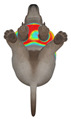
	Walking	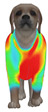	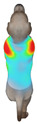	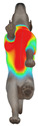	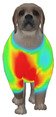	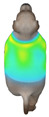	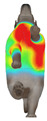
	Lying down	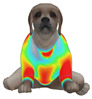	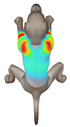	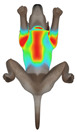	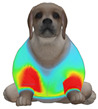	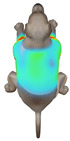	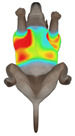
	Running	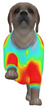	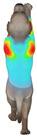	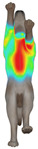	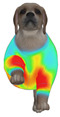	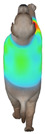	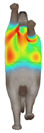
	Forelimb stretching	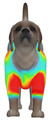	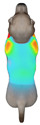	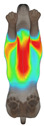	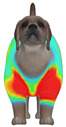	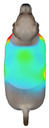	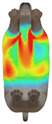
	Unilateral hind limb lifting	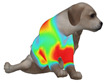	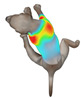	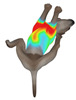	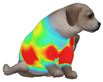	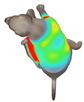	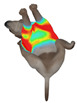

Note: Tables S5 and S6 provide the corresponding maps for the other two breeds.

**Table 6:** Clothing pressure data according to body condition (normal versus obese), posture, and fabric type obtained from virtual fitting simulations (unit: kPa).

Dog Breed	Posture	Fabric	P1 (Neck)		P2 (Chest)		P3 (Back)		P4 (Abdomen)	
			Normal	Obese	Normal	Obese	Normal	Obese	Normal	Obese
Bulldog	Walking	Cotton–spandex	8.54	9.43	16.37	18.33	6.14	7.28	18.08	20.99
		Polyester–spandex	9.82	10.97	18.82	21.53	7.06	8.62	20.79	24.76
		Polar fleece	11.94	13.64	22.92	26.79	8.57	10.94	25.34	31.26
	Sitting	Cotton–spandex	7.23	7.64	13.92	14.87	5.23	5.85	15.37	17.04
		Polyester–spandex	8.35	8.89	15.96	17.38	5.99	7.03	17.67	20.08
		Polar fleece	10.13	11.02	19.5	21.66	7.29	8.9	21.52	25.29
	Lying down	Cotton–spandex	6.88	7.28	13.66	14.56	4.85	5.47	14.92	16.52
		Polyester–spandex	7.93	8.47	15.69	17.04	5.55	6.54	17.16	19.48
		Polar fleece	9.63	10.5	19.11	21.2	6.79	8.25	20.87	24.53
	Running	Cotton–spandex	10.25	11.88	19.65	23.08	7.37	9.17	21.72	26.4
		Polyester–spandex	11.78	13.85	22.61	27.07	8.46	10.92	24.95	31.13
		Polar fleece	14.33	17.13	27.52	33.66	10.31	13.8	30.39	39.3
	Forelimb stretching	Cotton–spandex	9.55	10.9	18.57	21.41	6.81	8.35	19.88	23.82
		Polyester–spandex	11.06	12.7	21.33	25.07	7.85	9.84	22.88	28.04
		Polar fleece	13.38	15.79	25.98	31.2	9.54	12.47	27.88	35.32
	Unilateral hind limb lifting	Cotton–spandex	9.82	11.61	19.01	22.76	7.06	8.94	21.65	26.85
		Polyester–spandex	11.29	13.51	21.88	26.58	8.1	10.62	24.89	31.66
		Polar fleece	13.72	16.79	26.63	33.2	9.87	13.46	30.29	39.84
Labrador retriever	Walking	Cotton–spandex	9.13	9.74	18.77	21.13	6.99	8.39	20.85	24.36
		Polyester–spandex	10.47	11.31	21.58	24.76	8.05	9.91	23.95	28.8
		Polar fleece	12.79	13.8	26.26	30.84	9.79	12.6	29.16	36.3
	Sitting	Cotton–spandex	7.73	7.94	15.95	17.04	5.96	6.83	17.71	19.68
		Polyester–spandex	8.9	9.12	18.32	20.05	6.86	8.03	20.35	23.33
		Polar fleece	10.84	11.22	22.31	24.91	8.36	10.21	24.81	29.36
	Lying down	Cotton–spandex	7.38	7.51	15.6	16.79	5.52	6.34	17.19	19.13
		Polyester–spandex	8.45	8.71	17.94	19.6	6.38	7.53	19.75	22.59
		Polar fleece	10.33	10.67	21.87	24.4	7.76	9.5	24.07	28.49
	Running	Cotton–spandex	10.93	12.22	22.51	26.51	8.41	10.56	24.97	30.64
		Polyester–spandex	12.59	14.18	25.85	31.06	9.67	12.54	28.74	36.19
		Polar fleece	15.32	17.35	31.52	38.78	11.77	15.86	35.04	45.56
	Forelimb stretching	Cotton–spandex	10.25	11.22	21.24	24.56	7.8	9.58	22.94	27.51
		Polyester–spandex	11.75	12.99	24.42	28.81	8.96	11.41	26.37	32.54
		Polar fleece	14.34	15.94	29.75	35.87	10.9	14.42	32.11	41.08
	Unilateral hind limb lifting	Cotton–spandex	10.5	12.04	21.78	26.13	8.08	10.32	24.95	31.08
		Polyester–spandex	12.06	13.86	25.03	30.64	9.25	12.2	28.65	36.68
		Polar fleece	14.67	16.94	30.51	38.2	11.27	15.48	34.9	46.29
German shepherd	Walking	Cotton–spandex	9.64	10.42	20.11	21.92	7.87	9.34	22.41	24.79
		Polyester–spandex	11.09	12.11	23.15	25.43	9.05	11.05	25.81	28.82
		Polar fleece	13.52	14.87	28.18	31.43	11.02	13.96	31.41	35.76
	Sitting	Cotton–spandex	8.21	8.46	17.09	17.81	6.71	7.5	19.06	20.05
		Polyester–spandex	9.45	9.82	19.68	20.65	7.7	8.93	21.9	23.37
		Polar fleece	11.47	12.09	23.95	25.43	9.36	11.24	26.69	28.9
	Lying down	Cotton–spandex	7.78	8.06	16.78	17.44	6.23	6.96	18.49	19.46
		Polyester–spandex	8.97	9.31	19.3	20.22	7.14	8.31	21.25	22.64
		Polar fleece	10.9	11.4	23.48	24.95	8.73	10.44	25.89	28.03
	Running	Cotton–spandex	11.57	13.12	24.17	27.6	9.44	11.69	26.92	31.09
		Polyester–spandex	13.3	15.19	27.78	32.08	10.87	13.79	30.93	36.26
		Polar fleece	16.2	18.74	33.8	39.54	13.26	17.48	37.67	44.94
	Forelimb stretching	Cotton–spandex	10.84	12.07	22.82	25.58	8.78	10.66	24.66	28.02
		Polyester–spandex	12.46	13.95	26.21	29.65	10.05	12.6	28.39	32.64
		Polar fleece	15.18	17.14	31.91	36.64	12.26	15.93	34.56	40.46
	Unilateral hind limb lifting	Cotton–spandex	11.11	12.82	23.38	27.19	9.07	11.4	26.83	31.56
		Polyester–spandex	12.77	14.84	26.9	31.61	10.4	13.55	30.86	36.81
		Polar fleece	15.55	18.29	32.72	38.92	12.69	17.12	37.57	45.54

**Table 7:** ANOVA/GLM results for the effects of body condition, posture, fabric type, and breed on chest clothing pressure (P2) (kPa).

Source	df	SS	*F*	*p*-Value	Partial η^2^
Fabric	2	1358.35	2291.32	<0.001 ***	0.985
Body condition	1	299.37	1009.96	<0.001 ***	0.935
Posture	5	1684.43	1136.54	<0.001 ***	0.988
Breed	2	374.09	631.02	<0.001 ***	0.947
Body condition × fabric	2	18.79	31.69	<0.001 ***	0.475
Fabric × posture	10	37.34	12.60	<0.001 ***	0.753
Body condition × posture	5	63.24	42.67	<0.001 ***	0.643
Body condition × fabric × posture	10	2.27	0.77	0.661	0.099
Residual	70	20.75			

*** *p* < 0.001; df = degrees of freedom. Notes: partial η^2^ = SS_effect/(SS_effect + SS_error).

## Data Availability

All data supporting the findings of this study are available in the article and its Supplementary Materials. Due to licensing/copyright restrictions, the full CLO 3D project files cannot be publicly deposited; non-restricted materials and documentation are available upon reasonable request.

## Supplementary Materials

The following supporting information can be downloaded at: https://www.mdpi.com/article/10.3390/ani16071006/s1, Table S1. ANOVA/GLM results for the effects of body condition, posture, fabric type, and breed on neck clothing pressure (P1) (kPa); Table S2. ANOVA/GLM results for the effects of body condition, posture, fabric type, and breed on back clothing pressure (P3) (kPa); Table S3. ANOVA/GLM results for the effects of body condition, posture, fabric type, and breed on abdomen clothing pressure (P4) (kPa); Table S4. Landmark measurements used to standardize obese avatar generation (before/after and % change); Table S5. Qualitative clothing-pressure maps for the Bulldog across body condition (normal vs. obese), posture, and fabric type (front, top, and bottom views); Table S6. Qualitative clothing-pressure maps for the German shepherd across body condition (normal vs. obese), posture, and fabric type (front, top, and bottom views).

## Abbreviations

The following abbreviations are used in this manuscript:

BCS	Body condition score
ANOVA	Analysis of variance
GLM	General linear model
ROI	Region of interest
