# Supplementary material for: Comparative Analysis of Clothing Pressure Distribution in Obese and Normal-Weight Dogs Based on Material and Postural Variations Using CLO 3D Virtual Fitting
Source: Animals (Basel). 2026 Mar 25;16(7):1006. doi: 10.3390/ani16071006 (PMC13072379; doi:10.3390/ani16071006)
Supplement: Supplementary file 1 [file animals-16-01006-s001.zip › animals-4173088-supplementary.pdf]

Supplementary Table S1. ANOVA/GLM results for the effects of body condition, posture, fabric type, and breed on neck clothing pressure (P1) (kPa).

Source	df	SS	F	p-value	partial η^2
Fabric	2	301.73	4560.40	< 0.001***	0.992
Body condition	1	42.05	1271.10	< 0.001***	0.948
Posture	5	407.85	2465.70	< 0.001***	0.994
Breed	2	28.68	433.54	< 0.001***	0.925
Body condition × fabric	2	2.08	31.40	< 0.001***	0.473
Body condition × posture	5	12.41	75.04	< 0.001***	0.843
Fabric × posture	10	8.53	25.79	< 0.001***	0.787
Body condition × fabric × posture	10	0.37	1.11	0.366	0.137
Residual	70	2.32			

Supplementary Table S2. ANOVA/GLM results for the effects of body condition, posture, fabric type, and breed on back clothing pressure (P3) (kPa).

Source	df	SS	F	p-value	partial η^2
Fabric	2	234.89	1299.97	< 0.001***	0.974
Body condition	1	122.16	1352.10	< 0.001***	0.951
Posture	5	288.24	638.08	< 0.001***	0.979
Breed	2	96.47	533.90	< 0.001***	0.938
Body condition × fabric	2	9.75	53.98	< 0.001***	0.607
Body condition × posture	5	15.09	33.40	< 0.001***	0.705
Fabric × posture	10	7.07	7.83	< 0.001***	0.528
Body condition × fabric × posture	10	0.71	0.79	0.640	0.101
Residual	70	6.32			

Supplementary Table S3. ANOVA/GLM results for the effects of body condition, posture, fabric type, and breed on abdomen clothing pressure (P4) (kPa).

Source	df	SS	F	p-value	partial η^2
Fabric	2	1827.33	1565.50	< 0.001***	0.978
Body condition	1	611.56	1047.87	< 0.001***	0.937
Posture	5	2298.26	787.58	< 0.001***	0.983
Breed	2	459.66	393.80	< 0.001***	0.918
Body condition × fabric	2	44.17	37.84	< 0.001***	0.520
Body condition × posture	5	99.63	34.14	< 0.001***	0.709
Fabric × posture	10	53.35	9.14	< 0.001***	0.566
Body condition × fabric × posture	10	4.03	0.69	0.730	0.090
Residual	70	40.85			

Notes: partial η^2 *= SS_effect/(SS_effect + SS_error). *p < 0.05, **p < 0.01, ***p < 0.001.*

Supplementary Table S4. Landmark measurements used to standardize obese avatar generation (before/after and % change).

Breed	Neck N	Neck O	Δ	%Δ	Chest N	Chest O	Δ	%Δ	Abdomen N	Abdomen O	Δ	%Δ	Back length N	Back length O	Δ	%Δ
Bulldog	42.0	50.0	8.0	19.05%	68.0	85.0	17.0	25.00%	65.0	90.0	25.0	38.46%	38.0	38.0	0.0	0.00%
Labrador retriever	55.0	65.0	10.0	18.18%	78.0	100.0	22.0	28.21%	75.0	105.0	30.0	40.00%	55.0	55.0	0.0	0.00%
German shepherd	52.0	62.0	10.0	19.23%	80.0	102.0	22.0	27.50%	76.0	108.0	32.0	42.11%	58.0	58.0	0.0	0.00%

Notes: N = normal; O = obese. Units are cm. Back length was held constant during mesh modification; circumferential targets follow Table 1.

Supplementary Table S5. Qualitative clothing-pressure maps for the Bulldog across body condition (normal vs. obese), posture, and fabric type (front, top, and bottom views).

Fabric	Posture	Normal			Obese		
		Front	Top	Bottom	Front	Top	Bottom
Cotton–spandex	Sitting	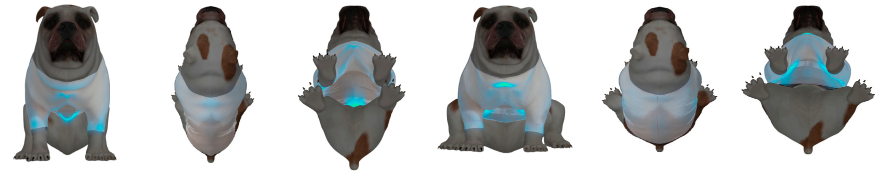					
	Walking	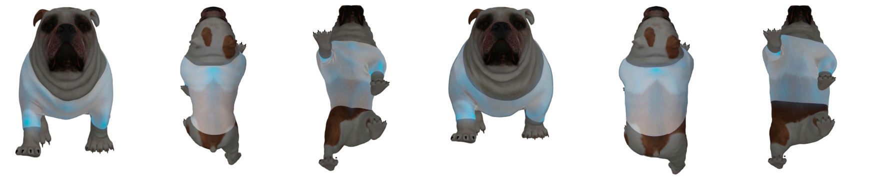					
	Lying down	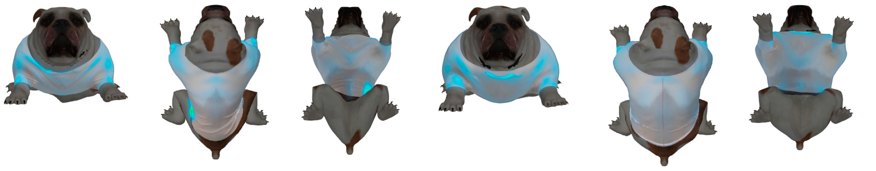					
	Running	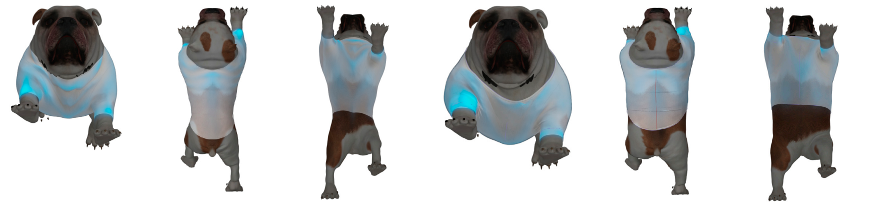					
	Forelimb stretching	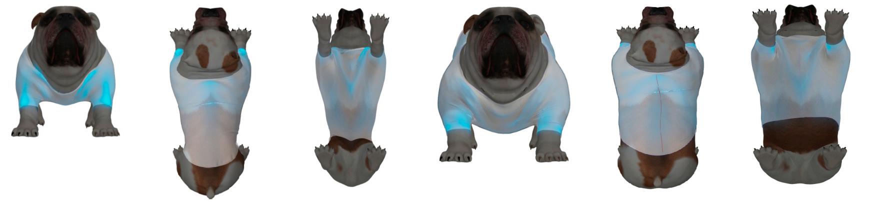					
	Unilateral hind limb lifting	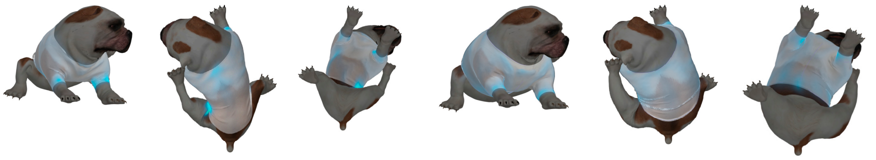					
Polyester–spandex	Sitting	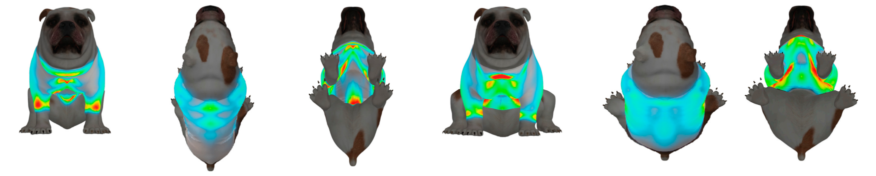					
	Walking	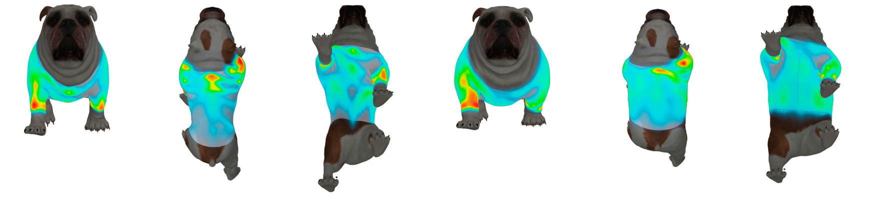					

	Lying down	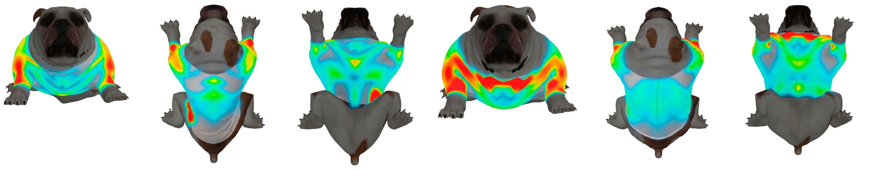
	Running	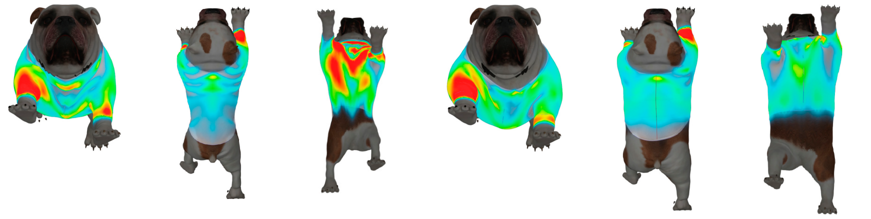
	Forelimb stretching	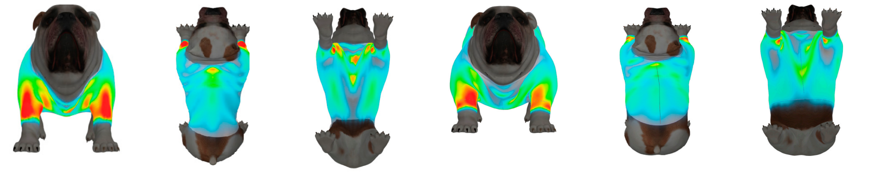
	Unilateral hind limb lifting	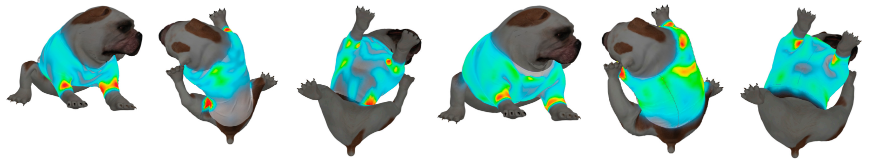
Polar fleece	Sitting	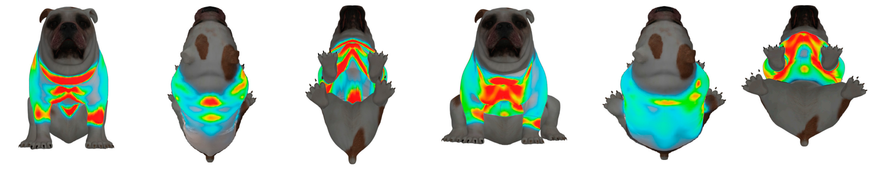
	Walking	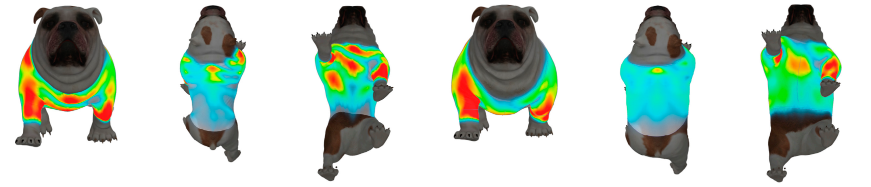
	Lying down	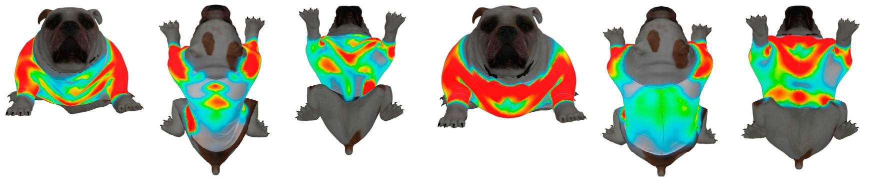
	Running	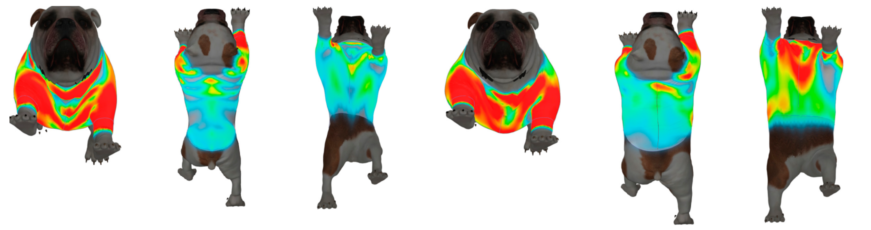

	Forelimb stretching	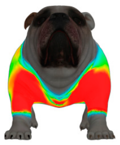	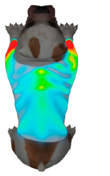	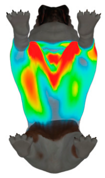	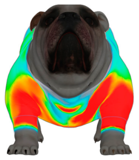	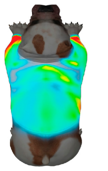	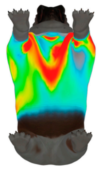
	Unilateral hind limb lifting	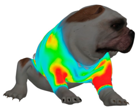	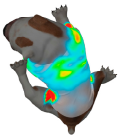	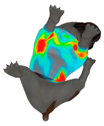	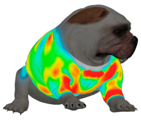	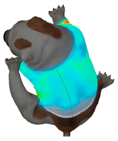	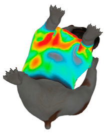

Supplementary Table S6. Qualitative clothing-pressure maps for the German shepherd across body condition (normal vs. obese), posture, and fabric type (front, top, and bottom views).

Fabric	Posture	Normal			Obese		
		Front	Top	Bottom	Front	Top	Bottom
Cotton–spandex	Sitting	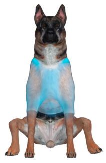	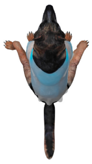	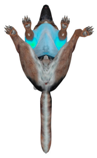	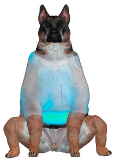	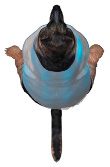	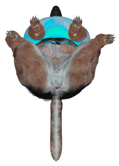
	Walking	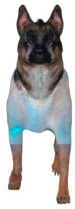	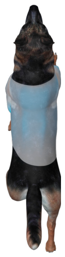	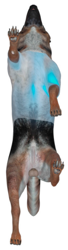	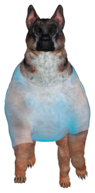	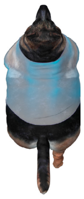	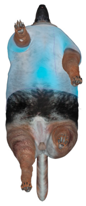
	Lying down	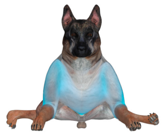	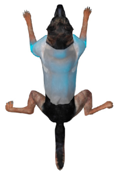	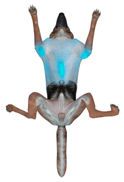	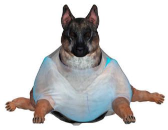	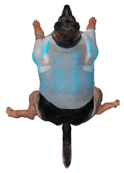	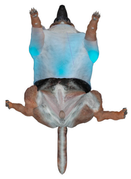
	Running	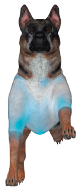	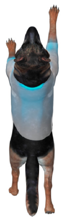	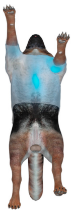	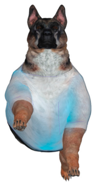	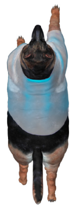	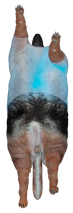

	Forelimb stretching	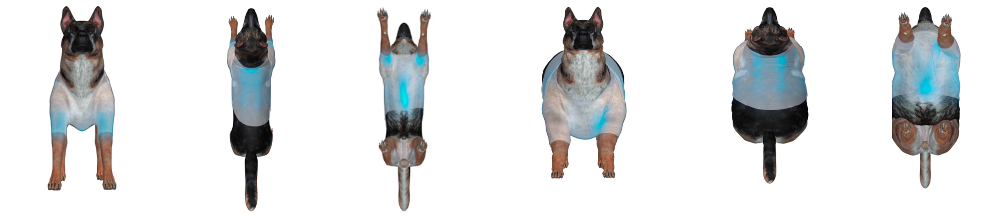
	Unilateral hind limb lifting	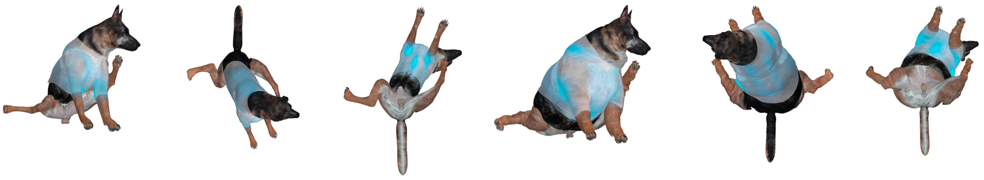
Polyester–spandex	Sitting	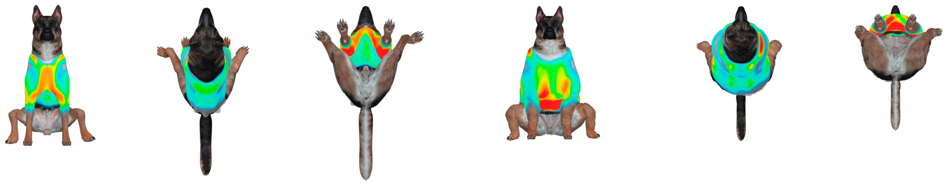
	Walking	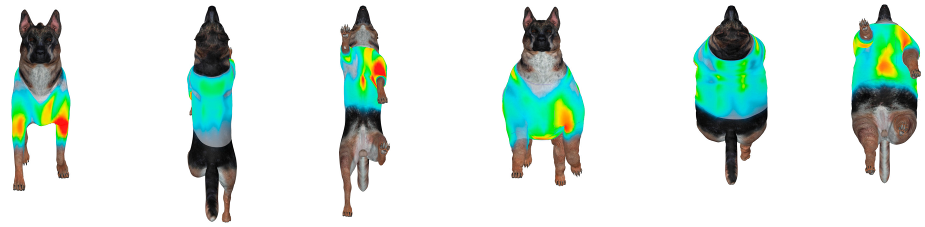
	Lying down	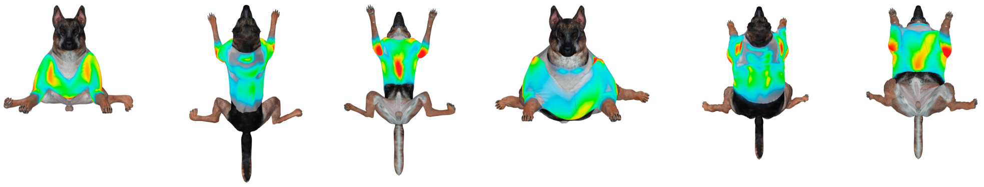
	Running	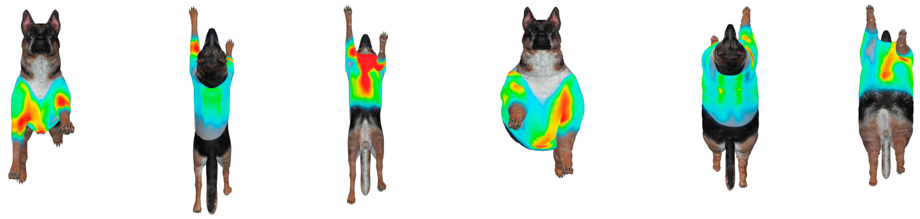
	Forelimb stretching	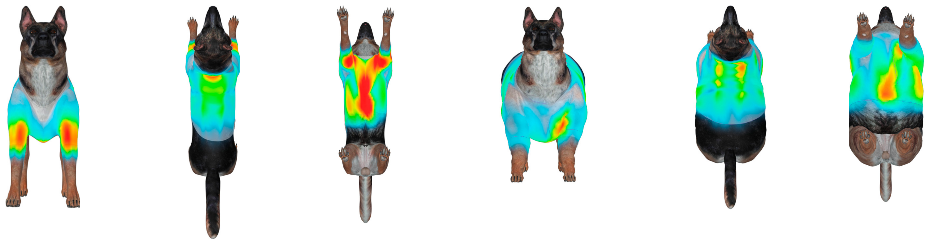

	Unilateral hind limb lifting	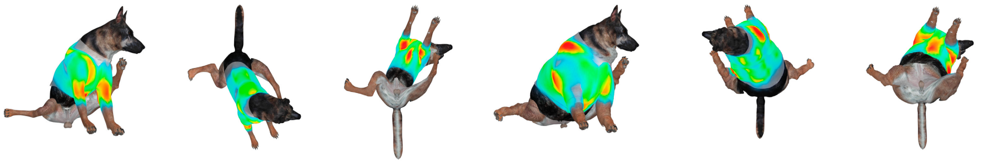
Polar fleece	Sitting	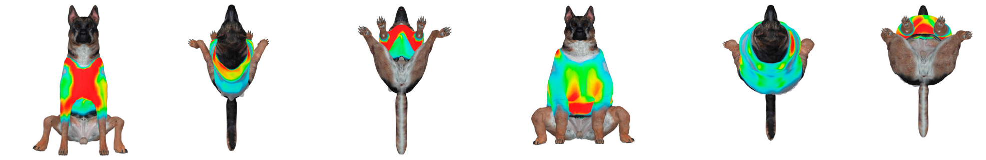
	Walking	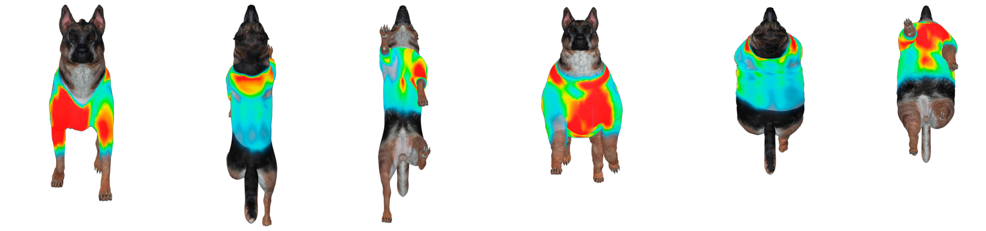
	Lying down	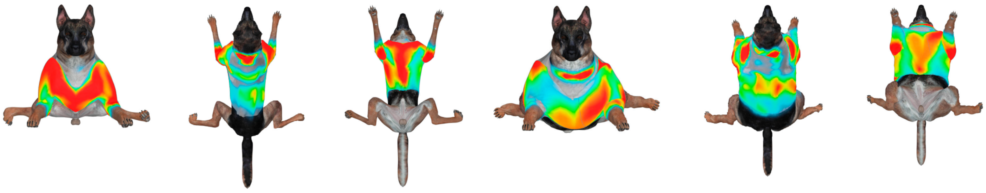
	Running	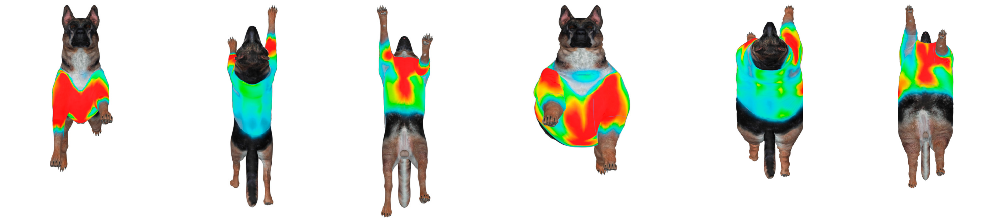
	Forelimb stretching	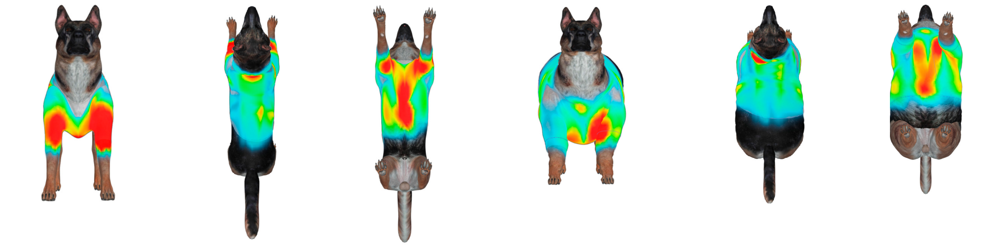
	Unilateral hind limb lifting	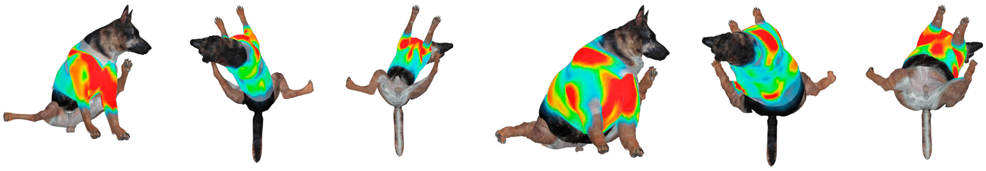
